# The Common Bean Small Heat Shock Protein Nodulin 22 from *Phaseolus vulgaris* L. Assembles into Functional High-Molecular-Weight Oligomers

**DOI:** 10.3390/molecules27248681

**Published:** 2022-12-08

**Authors:** Arline Fernández-Silva, Fernando Lledías, Jonathan Rodríguez-López, Juan E. Olivares, Leidys French-Pacheco, Marcela Treviño, Carlos Amero, Claudia Díaz-Camino

**Affiliations:** 1 LABRMN, Centro de Investigaciones Químicas, IICBA Universidad Autónoma del Estado de Morelos, Av. Universidad 1001, Colonia Chamilpa, Cuernavaca 62209, Mexico; 2 Instituto de Biotecnología, Universidad Nacional Autónoma de México, Av. Universidad 2001, Colonia Chamilpa, Cuernavaca 62210, Mexico; 3 School of Pure and Applied Sciences, Florida SouthWestern State College, Fort Myers, FL 33919, USA

**Keywords:** small heat shock proteins, chaperones, *Phaseolus vulgaris* L., heat stress, oxidative stress

## Abstract

Small heat shock proteins (sHsps) are present in all domains of life. These proteins are responsible for binding unfolded proteins to prevent their aggregation. sHsps form dynamic oligomers of different sizes and constitute transient reservoirs for folding competent proteins that are subsequently refolded by ATP-dependent chaperone systems. In plants, the sHsp family is rather diverse and has been associated with the ability of plants to survive diverse environmental stresses. Nodulin 22 (*Pv*Nod22) is an sHsp of the common bean (*Phaseolus vulgaris* L.) located in the endoplasmic reticulum. This protein is expressed in response to stress (heat or oxidative) or in plant roots during mycorrhizal and rhizobial symbiosis. In this work, we study its oligomeric state using a combination of in silico and experimental approaches. We found that recombinant *Pv*Nod22 was able to protect a target protein from heat unfolding in vitro. We also demonstrated that *Pv*Nod22 assembles into high-molecular-weight oligomers with diameters of ~15 nm under stress-free conditions. These oligomers can cluster together to form high-weight polydisperse agglomerates with temperature-dependent interactions; in contrast, the oligomers are stable regarding temperature.

## 1. Introduction

All living organisms have evolved mechanisms to cope with the damaging effects of high temperatures and other proteotoxic stresses. One of these mechanisms involves the action of small heat shock proteins (sHsps), an archaic and ubiquitous class of ATP-independent chaperones that associate with misfolded proteins [1]. The folding-competent proteins held in these transient reservoirs are subsequently refolded by ATP-dependent chaperone systems. While sHsps are found in all domains of life, members of the sHsp family in plants are quite diverse and abundant.

Plant sHsps are classified into subfamilies based on sequence similarity and cellular localization. Subfamilies include proteins targeting the cytosol, nucleus, endoplasmic reticulum, chloroplasts, mitochondria, and peroxisomes [2,3]. The large expansion of the sHsp classes is associated with early diversification and is thought to reflect the ability of plants to survive diverse environmental stresses [4,5]. Indeed, while a few plant sHsps are expressed constitutively, the expression of most plant sHsps only occurs under environmental stress or during some stages of growth and development, such as embryogenesis, germination, the development of pollen grains, and fruit ripening [6]. Although there is no clear explanation of the specific molecular function of plant sHsps beyond heat acclimation, some studies have shed light on their role in maintaining protein stability [7], protein translocation [8], or the protection of cell membranes [9,10].

sHsps have molecular masses between 12 and 24 kDa and are characterized by the presence of a conserved central alpha crystallin domain (ACD) flanked by an N-terminal domain (NTD) and a C-terminal domain (CTD) [4,5,11]. The ACD consists of a β-sandwich fold composed of two antiparallel sheets, while the NTD and CTD terminal extensions vary in length and tend to be more divergent and flexible [12,13,14].

Most sHsps are known to exist in their native state as multi-subunit high-molecular-weight (HMW) oligomers; however, the resulting complexes differ in their total number of subunits [5]. For instance, the mitochondria sHsp22 of pea (*Pisum sativum*) tends to form 8-mers, 12-mers or 20-mers [15], and the chloroplast sHsp22.3 of sugarcane (*Saccharum* spp.) forms complexes of 16 subunits [16], whereas the cytosolic sHsp16.9 of wheat (*Triticum aestivum* L.) forms double rings of 12 subunits or tetrahedral dodecamers [17,18]. Acquiring high-resolution structural data for the oligomeric forms of plant sHsps has been challenging due to the tendency to polydispersity and dynamics [4,19]. The only high-resolution oligomeric structures currently available from plant sHsps are those of sHsp16.9 from *Triticum aestivum* L. [17].

Given that the oligomeric state of some sHsps is affected by environmental conditions such as temperature and pH, it has been proposed that when these conditions fluctuate, some sHsps disassemble into smaller subunits to expose clusters of hydrophobic residues that bind to the hydrophobic patches of misfolded proteins, forming stable complexes with them and thereby preventing their irreversible aggregation [1,15,18,20,21]. Nevertheless, some other sHsps have been reported to be fully functional in their oligomeric state, showing the ability to suppress client protein aggregation under stress conditions [18,22]. Therefore, it is becoming increasingly clear that no single model is sufficient to describe the structure, function, and mechanism of action for all sHsps.

Nodulin 22 (*Pv*Nod22) is an sHsp from common bean (*Phaseolus vulgaris* L.) which is highly expressed in plant roots during mycorrhizal and rhizobial symbiosis [23], and in the foliage in response to heat or oxidative stress [24]. *Pv*Nod22 is localized in the endoplasmic reticulum (ER), and recent findings from our group suggest that its chaperone activity is linked to the unfolded protein response (UPR) [25]. The UPR is a cellular reaction to stress that modulates the capacity and quality of the polypeptide-folding process in the ER, thus minimizing the cytotoxic impact of malformed proteins. This response has a fundamental role in the adaptation and survival of plants regarding stress; we have proposed the *Pv*Nod22 function to be relevant in the maintenance of protein homeostasis [24].

The identification of phenotypes associated with specific plant sHsps has been challenging due to the coexistence of multiple sHsps from the same class in any given species, along with the functional redundancy observed in single gene knockouts [26]. This poses a major constraint to understanding the biological functions of these proteins. Interestingly, we found that the silencing of *Pv*Nod22 expression resulted in both foliar necrosis and growth inhibition of plants cultivated under optimal conditions, indicating that *Pv*Nod22 plays an essential role for the plant [24,27]. In addition, the reduced expression of this protein in the transgenic roots of common bean plants during symbiosis diminished their rhizobial infection levels, indicating that *Pv*Nod22 function is also involved in this ancient process [25].

Given the dynamic structural changes that are characteristic of sHsps oligomers and which are likely related to their functional role, in this work we use in silico, biochemical and biophysical methods to better understand the functional oligomeric states of *Pv*Nod22. Our results show that *Pv*Nod22 assembles into high-molecular-weight oligomers, which are stable within a temperature range of 25–60 °C, and tend to cluster together in solution.

## 2. Results

### 2.1. Bioinformatic Analysis and Molecular Modeling of PvNod22

**Figure 1:**
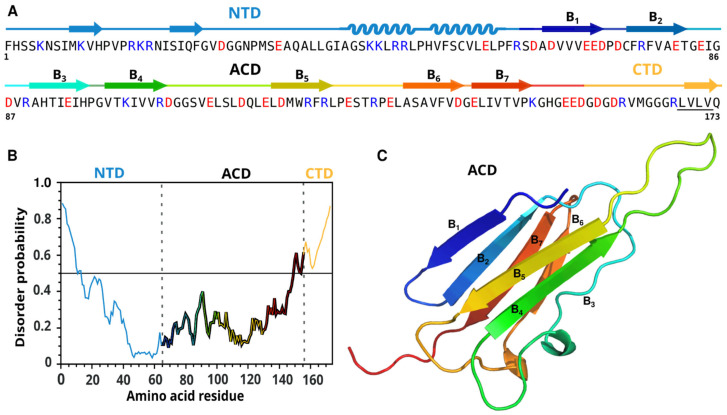
Sequence analysis and molecular modeling of *Pv*Nod22. (**A**) *Pv*Nod22 amino acid sequence. Negatively and positively charged amino acids are colored in red and blue, respectively. Secondary structure prediction is indicated for all the domains: blue (NTD), rainbow (ACD) and orange (CTD). The I/V-X-I/V motif in the CTD region is underlined. (**B**) Prediction of intrinsically unstructured regions of *Pv*Nod22 using IUPred based on estimated energy content, which shows that both the N- and C-terminal extensions (blue and orange) of *Pv*Nod22 have disorder probability values above the threshold (score: 0.5), while the central domain is ordered (rainbow). (**C**) ACD homology model using the crystallographic structure of TasHsp16.9 (PDB code 5DS1) as a template. The seven predicted β-sheets are shown.

*Pv*Nod22 consists of 173 residues and has a molecular weight of 21.6 kDa. Sequence analysis shows that *Pv*Nod22 is composed of a central alpha-crystalline domain (ACD, residues 65 to 154) (Figure S1) flanked by an N-terminal segment of 64 residues and a short C-terminal region of 19 residues. These results are consistent with the structural features of sHsps, which have an ACD domain flanked by two external domains (the NTD and the CTD) (Figure 1). Neither the NTD nor the CTD regions share sequence homology with any known protein structure. The NTD was predicted to have mostly beta-sheets and alpha-helix secondary structure elements, while the CTD was predicted to be highly disordered (Figure 1).

The ACD region of *Pv*Nod22 has a sequence identity of 32.1% with the class I sHsp 18.1 from *Pisum sativum*, and of 30.6% with *Triticum aestivum* sHsp16.9 (Figures S1 and S2). We constructed homology models using both templates, and the homology models were compared with the predicted AlphaFold structure [28]; the results were very similar (Figures S2 and S3). According to the homology models, *Pv*Nod22-ACD is composed of seven β-sheets linked together by six loops of variable lengths (Figure 1). Taking advantage of the quaternary prediction of SWISS MODELER, we also investigated the putative dimer. Interestingly, *Pv*Nod22 appears to have a shorter region between β5 and β6, which, for the two homologous proteins, would correspond to β7. This sheet in these two proteins made inter-molecular interactions in the dimer (Figure S2).

### 2.2. PvNod22 Has a Chaperone Function In Vitro

Previous studies from our laboratory have shown the accumulation of the *Pv*Nod22 transcript in planta during heat stress, as well as the ability of the purified protein to facilitate the in vitro refolding of a model protein [24,25].

**Figure 2:**
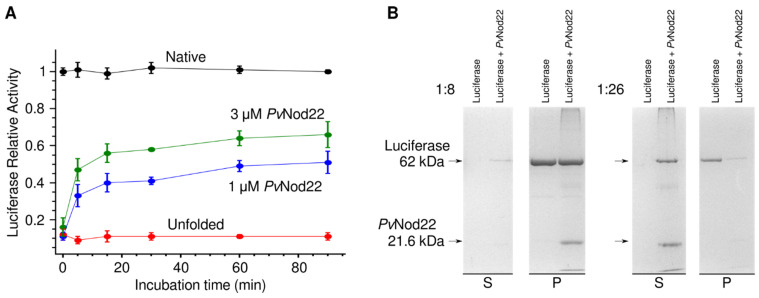
*Pv*Nod22 displays in vitro chaperone activity. (**A**) Ability of *Pv*Nod22 to suppress luciferase aggregation. Luciferase was incubated at 42 °C in the absence or presence of 1 or 3 µM *Pv*Nod22. After that, luciferase was incubated in a refolding solution. The activity was measured for 90 min, and the native protein was used as a control. (**B**) Either 3 or 1.5 µM firefly luciferase was mixed in PBS in the absence or presence of 40 µM *Pv*Nod22 (1:8 or 1:26 luciferase: *Pv*Nod22 molar ratio, respectively), incubated at 42 °C and centrifuged. Soluble (S) and pellet (P) fractions were analyzed by 15% (*w*/*v*) SDS-PAGE using Coomassie blue staining.

Here, we tested the ability of *Pv*Nod22 to suppress the temperature-induced aggregation of firefly luciferase in vitro. In these assays, luciferase was heat-denatured for 20 min at 42 °C with or without *Pv*Nod22. Samples were then shifted to a solution containing ATP-dependent chaperones, and the luciferase activity recovery was measured at specific timepoints. As shown in Figure 2A, the activity of non-heated (native) luciferase remains constant during the assay. In contrast, the activity of luciferase was reduced to less than 10% after heating at 42 °C for 20 min (unfolded). We observed a concentration-dependent increase in the recovery of the relative luciferase activity in the presence of *Pv*Nod22. These observations are consistent with the model in which sHsps stabilize heat-sensitive target proteins exposed to high temperatures by keeping them in a soluble state, and therefore competent for refolding [3].

Then, we tested the ability of *Pv*Nod22 to protect luciferase from heat-induced insolubilization. Aliquots from luciferase incubated at 42 °C with and without *Pv*Nod22 were analyzed by SDS-PAGE. As shown in Figure 2B, luciferase by itself undergoes aggregation at 42 °C; thus, it is completely absent from the supernatant and is exclusively found in the pellet fraction. The presence of *Pv*Nod22 at a 1:8 luciferase: *Pv*Nod22 molar ratio prevented the precipitation of only a small fraction of luciferase, as indicated by the fact that only a small amount of this protein is present in the supernatant, and most of it is found in the pellet fraction. Conversely, an almost complete suppression of the heat-induced precipitation of luciferase was observed when the molar ratio was increased to 1:26 (Figure 2B). In this case, most of the luciferase is found in the supernatant fraction, and only a small amount remains in the pellet. The results obtained with other tested molar ratios below 1:26 were consistent, showing a greater suppression of the temperature-induced aggregation of luciferase as the proportion of *Pv*Nod22 was increased.

Incidentally, while *Pv*Nod22 by itself does not precipitate at temperatures below 70 °C, this protein was consistently found in the pellet fraction whenever it was combined with luciferase at 42 °C, in a seemingly proportional amount to that of denatured luciferase (Figure 2). This indicates that, while being incapable of completely preventing luciferase aggregation at lower molar ratios, *Pv*Nod22 does bind to the denatured protein.

### 2.3. PvNod22 Exists as HMW Oligomers under Non-Stress Conditions

Crosslinking with glutaraldehyde was used to further investigate the oligomeric state of *Pv*Nod22. Aliquots from the cross-linking reaction were quenched at various incubation timepoints and analyzed by SDS-PAGE (Figure S4). Our results indicate that HMW complex *Pv*Nod22 oligomers are stabilized immediately after incubation with glutaraldehyde, with no further changes observed at longer incubation times. This suggests that *Pv*Nod22 exists in the aqueous solution as HMW oligomers in non-stressed conditions.

**Figure 3:**
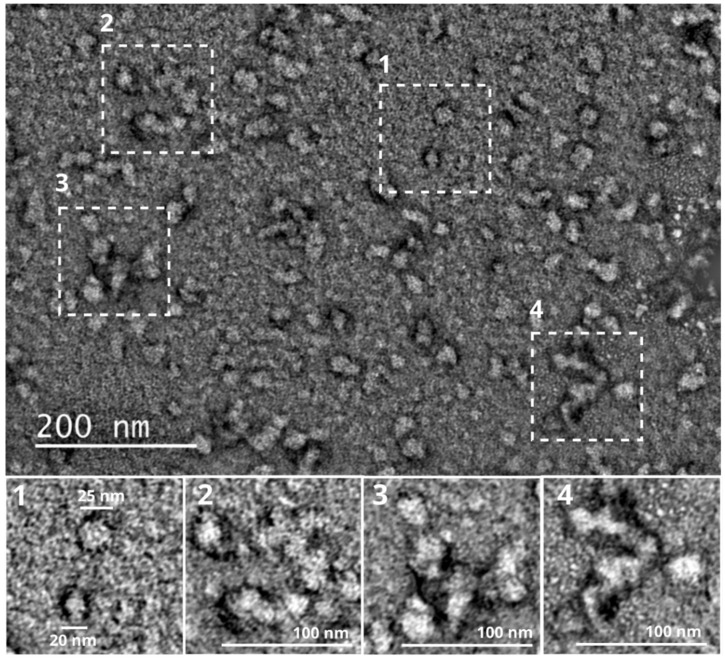
*Pv*Nod22 forms oligomers that cluster together in non-stressed conditions. Negative stain TEM micrograph of *Pv*Nod22 at room temperature. Areas labeled 1–4 are enlarged and are shown as insets. The samples were under conditions similar to those used in the experiment presented in Figure 2A, in which *Pv*Nod22 is functional.

To analyze the structures of the oligomeric complexes in more detail, we used transmission electron microscopy (TEM). Images of negatively stained *Pv*Nod22 samples revealed the presence of polydisperse HMW oligomers, which vary in size and shape (Figure 3). Intriguingly, such oligomers are often observed to be clustered together, forming larger agglomerates of varying structures (Figure 3).

### 2.4. Dynamics of Recombinant PvNod22 Oligomeric States under Stress Conditions

**Figure 4:**
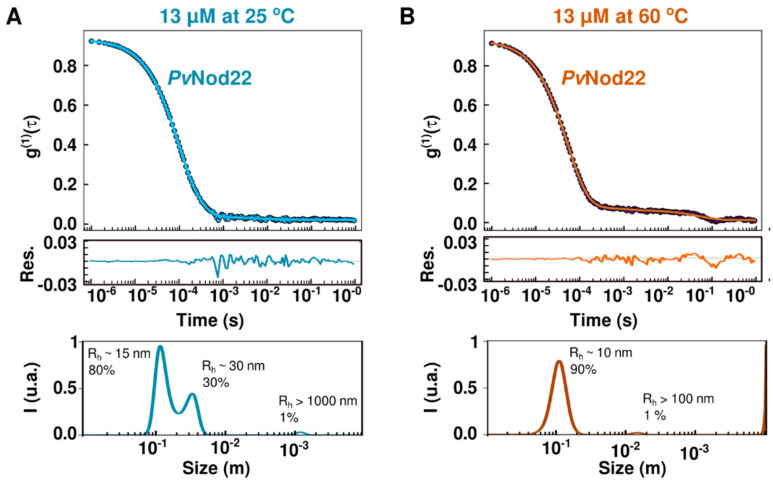
The oligomeric state of *Pv*Nod22 is affected by temperature. (**A**) DLS measurements of 13 µM *Pv*Nod22 at 25 °C. (**B**) DLS measurements of 13 µM *Pv*Nod22 at 60 °C. The shift in the correlation curve from right to left indicates that the hydrodynamic radius of the particle decreases as the temperature increases. The residual for the fittings and the corresponding size distribution are shown in both panels.

To better understand the inter-converting polydisperse ensembles of oligomeric states, we examined the temperature and protein concentration dependence of the *Pv*Nod22 oligomeric particle size by dynamic light scattering (DLS). While it is challenging to correlate the diffusion coefficient (D) of the particles present in a polydisperse sample with their absolute size, DLS measurements are useful for revealing changes in the oligomeric conformation of a protein. Initially, measurements were conducted using 13 µM *Pv*Nod22 in PBS pH 7.5 at 25 °C. The DLS data show polydisperse curves, from which we obtain one major component with a diffusion coefficient of D = 1.7 × 10^−8^ cm^2^/s, which would correspond to a spherical particle with an apparent hydrodynamic radius (R_h_) of 15 nm (Figure 4A). We also detected other small populations of large particle sizes that could correspond to the agglomerates of oligomers. These measurements are in agreement with the polydisperse state observed with electronic microscopy.

Measurements at higher temperatures (from 25 to 60 °C) yielded a shift in the decay rates of scattered light (correlation function), indicating that the overall size of the HMW *Pv*Nod22 oligomers decreases as the temperature increases (Figure 4B and Figure S5). At 60 °C, the major component corresponds to a particle with an apparent R_h_ of ~10.4 nm, which may represent the smaller oligomers observed with microscopy. Interestingly, similar particle sizes were observed at lower *Pv*Nod22 concentrations (3.2, 2.4 and 1.8 µM) (Figure S5), indicating that protein concentration does not significantly affect oligomer size in this concentration range. Together, our DLS data show that *Pv*Nod22 is polydisperse in solution and exists as HMW oligomers even at high temperatures.

### 2.5. PvNod22–PvNod22 Interactions

**Figure 5:**
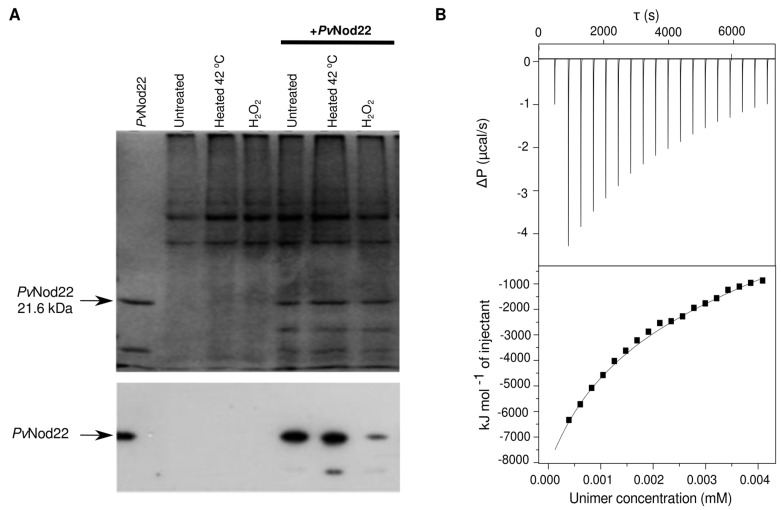
*Pv*Nod22 oligomers interact with each other. (**A**) Protein profile on a Coomassie-stained 15% SDS-PAGE gel of collected fractions eluted from the *Pv*Nod22 affinity column at 300 mM imidazole. Lane 1: Purified *Pv*Nod22 bound to the Ni-NTA resin. Lanes 2 to 4: Plant proteins from the common bean foliar extracts applied that are able to bind to the Ni-NTA matrix in the absence of *Pv*Nod22: heated foliar tissues at 42 °C for 30 min, treated foliar tissues with 1 mM hydrogen peroxide. Lanes 5 to 7: Plant extract proteins bound to the Ni-NTA-*Pv*Nod22 affinity column from untreated foliar tissues, heated at 42 °C for 30 min and treated with 1 mM hydrogen peroxide protein extracts. Bottom panel: Western blot analysis of the same samples using the anti-*Pv*Nod22 antibody. (**B**) Representative thermogram from an ITC dilution experiment with recombinant *Pv*Nod22. The top graph is the differential potential after each *Pv*Nod22 injection (2 µL from a 13 µM solution, with 180 s intervals between injections). The bottom graph is the integrated injection heat values, correcting for the heat of the buffer control. The enthalpy and dissociation constants obtained were ΔH = −4.96 × 10^4^ ± 2.34 × 10^2^ kJ mol^−1^ and a Kd of 9.13 ± 1 µM, respectively.

Taking advantage of the N-terminal histidine tag in the recombinant *Pv*Nod22, we investigated the ability of the oligomer *Pv*Nod22 protein to interact with plant proteins from leaf extracts. We prepared a *Pv*Nod22 affinity column by immobilizing the recombinant protein into an Ni-NTA resin; then, we incubated this matrix with foliar protein extracts subjected either to heat shock (42 °C) or oxidative stress (H_2_O_2_). Figure 5A shows the interaction among individual *Pv*Nod22 monomers; this interaction seems responsible for the formation of the large agglomerates, as detected in TEM, cross-linking and DLS experiments.

Isothermal titration calorimetry (ITC) dilution experiments were used to gain insight into the thermodynamics of *Pv*Nod22–*Pv*Nod22 interaction. Serial injections of *Pv*Nod22 into the cell were performed to determine the disassociation enthalpy (ΔH) and the disassociation constant (Kd) required for the breaking apart of *Pv*Nod22 clusters. The results show that *Pv*Nod22 agglomerates disassociate in a single exothermic transition (Figure 5B), indicating that *Pv*Nod22 oligomer–oligomer dissociation involves favorable enthalpy (ΔH = −4.96 × 10^4^ ± 2.34 × 10^2^ kJ/mol). Consequently, the complex formation should be entropically driven, which could be mediated by the predicted disordered CTD and by the water molecules released from the interface that increase their translational and rotational movements.

## 3. Discussion

Along with other molecular chaperones, sHsps protect cellular proteins from stress-induced damage by an early association with misfolded proteins and by suppressing their nonspecific aggregation [1]. Members of the sHsp family have diverse functions including stress tolerance, protein folding, protein degradation and signal transduction, among others [5,29]. In bean cv. *Negro Jamapa*, the transcription of *Pv*Nod22 is induced in response to symbiosis, heat stress, or oxidative stress [23,24]. We have previously shown that *Pv*Nod22 function is part of the unfolded protein response; thus, Nod22 appears to be an ER-resident sHsp [25].

Bioinformatic analyses were consistent with *Pv*Nod22 being composed of N- and C-terminal domains and a central ACD. According to the generated homology model, the *Pv*Nod22-ACD is composed of seven beta sheets. Interestingly, the homology proteins used as templates to make this model are composed of eight beta sheets. The region containing β6 for the other structures is shorter for *Pv*Nod22, and, therefore, it seems unlikely that the interaction described for other sHsps can be formed, in which the β6 strand interacts with the β2 of another subunit. The CTD was predicted to be primarily disordered; this region contains the conserved I/V-X-I/V motif found in virtually all eukaryotic sHsps (Figure 1A, underlined sequence). This motif has been reported to bind to the hydrophobic groove formed by the β4 and β8 strands of a subunit in the neighboring dimer [30].

Cross-linking experiments and functional analysis suggest the existence of HMW *Pv*Nod22 oligomers in solution, while TEM revealed that *Pv*Nod22 exists as polydisperse oligomers (Figure 3). Multiple studies have shown that sHsps display dynamic polydispersity, which involves the continual recycling of subunits [19,31] For instance, pea HspP22 shifts from 20-mers to 12-mers to 8 mers [15], while the dodecamer of pea Hsp18.1CI is in equilibrium with dimers, monomers and higher-order oligomers in solution [19]. Consistent with this, TEM images show particles of different shapes and sizes, ranging from ~10 nm in diameter to approximately 30 nm. Moreover, it seems that these oligomers cluster together to form larger agglomerates.

Different heat-induced activation mechanisms have been proposed for sHsp; one of the most accepted mechanisms is the dissociation of oligomers into smaller species. Therefore, we examined whether the structure of *Pv*Nod22 could be affected by a temperature increase from 25 °C to 60 °C, which corresponds to severe heat stress conditions for most plants. DLS experiments show that while *Pv*Nod22 forms polydisperse HMW oligomers in the solution with a predominant particle size of ~15 nm in diameter at 25 °C (Figure 4), the particle size changes to ~10 nm at 60 °C. It is possible that we may be detecting a shift in populations from the largest to the smallest particles seen through the microscope. While the changes in concentrations did not result in significant changes in size. These results show that *Pv*Nod22 oligomers are very stable and do not easily disassemble into smaller subunits, even at low concentrations and high temperatures.

sHsps display not only diversity conformation but also a remarkable degree of functional structural variation. For instance, the functional form of Rice Hsps is as dimers [32]. Likewise, Santhanagopalan and colleagues demonstrated that Ta Hsp 16.9 and Ps Hsp 18.1 interact with their client proteins only in dimeric form [18]. However, other sHsps can recognize and refold their target proteins in an oligomeric state. For instance, the sHsp Xa HspA isolated from a plant pathogen forms a 36 mer in solution, and this oligomer can interact with target proteins. Similarly, both the Hsp18.1 isolated from *P. sativum* and the CeHsp17 isolated from *C. elegans* are activated only in the oligomeric state and in super molecular assemblies, respectively [19]. *Pv*Nod22 appears to belong to this group and to function in its oligomeric state.

Intriguingly, TEM images show that while *Pv*Nod22 exists as individual HMW oligomers, these may be found to be clustered together, forming agglomerates of variable sizes and shapes (Figure 3). These *Pv*Nod22–*Pv*Nod22 interactions were confirmed using a column with a recombinant oligomer *Pv*Nod22 and foliar extracts subjected to heat (Figure 5A). To further investigate the formation of these large agglomerates, we used calorimetry. ITC dilution experiments showed that the dissociation of HMW *Pv*Nod22 agglomerates into smaller oligomeric forms is a process accompanied by a favorable enthalpy (Figure 5B). This enthalpy is most likely due to an increase in ordered water molecules to solvate the smaller oligomers during the disassembly of high-order *Pv*Nod22 agglomerates, resulting in a positive enthalpy and negative entropy cost. The low apparent K_a_ that we obtained suggests a moderate affinity among the oligomers, allowing for an equilibrium between assembled and disassembled agglomerates. Based on these results, *Pv*Nod22 seems to fold into stable oligomers that cluster to form larger agglomerates of varying structures. A similar cluster formation has been previously reported for other sHsps [33,34,35].

All the results suggests that the mechanism of action is the following: *Pv*Nod22 exists in a wide variety of oligomers with different sizes, which tend to agglomerate. In response to stress, *Pv*Nod22 would capture aggregation-prone partially unfolded proteins and form larger complexes, thus preventing irreversible aggregation. The substrates in the complexes would then be released for refolding with the help of ATP-dependent molecular chaperones during the recovery from stress.

## 4. Materials and Methods

### 4.1. Expression and Purification

*Escherichia coli* XL1-Blue cells were transformed with pQE30-*Pv*Nod22 [23] to obtain a recombinant *Pv*Nod22 containing a 6xHis-tag at the N-terminus and lacking the ER-signal peptide (i.e., the first 25 amino acid residues). Transformed *E. coli* cells were grown at 37 °C with agitation at 200 rpm in 1 L of Luria Bertani broth medium containing 100 μg/mL ampicillin until the OD_600_ reached 0.6. Protein expression was then induced with 1 mM isopropyl 1-thio-D-galactopyranoside. Four hours later, the cells were harvested by centrifugation (8000× *g* at 4 °C for 15 min), resuspended in 25 mL of 100 mM PBS (4.3 mM Na_2_HPO_4_, 1.4 mM KH_2_PO_4_, 100 mM NaCl, pH 7.5), 10% (*v*/*v*) glycerol, 0.5% (*v*/*v*) Tween 20, 1 mM aminocaproic acid and 1 mM phenyl methyl sulfonyl fluoride supplemented with 100 mg/mL lysozyme and incubated at 4 °C for 30 min. The resulting cell lysate was centrifuged (10,000× *g* at 4 °C for 15 min). The pellet was resuspended in 25 mL of the same buffer, washed and resuspended again and then sonicated six times for 20 s with intervals of 20 s of rest and centrifuged (10,000× *g* at 4 °C for 15 min).

Since *Pv*Nod22 is expressed in *E. coli* as inclusion bodies, the pellet was washed with 1 M urea, 2% (*v*/*v*) Triton X-100 and centrifuged (10,000× *g* at 4 °C for 30 min). The pellet was solubilized in denaturing buffer (20 mM PBS, pH 7.5, 8 M urea), stirred for 3 h at room temperature and centrifuged (10,000× *g* at room temperature for 30 min). Solubilized recombinant *Pv*Nod22 was bound to an Ni-NTA agarose resin pre-equilibrated in denaturing buffer by batch-absorption overnight at room temperature. *Pv*Nod22 refolding and purification were carried out under gravity. Briefly, B-cyclodextrin resin was packed into a 2.5 cm diameter plastic syringe and washed with five column volumes (CV) of the denaturing buffer containing 20 mM imidazole and 10 mM of 2-mercaptoethanol. Next, the column was washed with 10 CV of PBS, pH 7.5, containing 0.1% Triton X-100 and 500 mM NaCl. This was followed by a wash with 10 CV of PBS, pH 7.5 containing 5 mM β-cyclodextrin to allow the *Pv*Nod22 to refold. An additional wash with PBS was applied to remove the remaining β-cyclodextrin before elution. Refolded *Pv*Nod22 was eluted with PBS pH 7.5 supplemented with 300 mM imidazole. Recovered fractions were extensively dialyzed for two days with PBS pH 7.5 at 10 °C, and protein purity was evaluated by 12% (*w*/*v*) SDS-PAGE. As a control ensuring that the proteins were well folded and functional, anti-precipitation activity assays were measured.

### 4.2. Anti-Precipitation Activity Assay

We tested the ability of recombinant *Pv*Nod22 to inhibit client protein aggregation at a high temperature by measuring the recovery activity with a luciferase activity assay and SDS-PAGE gels. First, 1 µM luciferase (Promega, Madison, WI, USA) was incubated for 20 min at 42 °C in the absence or presence of either 1 or 3 µM *Pv*Nod22 in 25 mM HEPES/KOH (pH 7), 10 mM KCl, 5 mM MgCl_2_ and 2 mM dithiothreitol. Then, the luciferase was incubated in a solution containing 30 μL of nuclease-treated rabbit reticulocyte lysate at 30 °C to refold the protein. The luciferase recovery activity was measured at different times (5 to 90 min) in a luminometer (Monolight 3010, BD Biosciences, San Jose, CA, USA). The activity of the unheated sample was used as a control. Data points and associated error bars represent three independent *Pv*Nod22 purifications. Additionally, either 5 or 1.5 µM of luciferase were suspended in 20 μL of PBS pH 7.5 and incubated at 42 °C in the absence or presence of 40 µM *Pv*Nod22 (1:8 and 1:26 molar ratios of luciferase: *Pv*Nod22, respectively) for 20 min. Then, the solution was incubated for 1.5 h at 30 °C, and protein aggregates were recovered by centrifugation (16,000× *g* for 5 min). The proteins present in the supernatants and pellets were analyzed by 15% (*w*/*v*) SDS-PAGE.

### 4.3. In Silico Modeling and Identification of Intrinsically Disordered Regions in the Protein

The amino acid sequence of *Pv*Nod22 was taken from UniprotKB [36]. Sequence alignments were performed using the “Align tool” [37], and secondary structure prediction was performed using Psipred [38]. Additionally, the IUPred server [39] was used to predict potential unfolded regions in the protein.

A homology monomer model of the conserved *Pv*Nod22 ACD region was generated using the crystal structure of *Triticum aestivum* sHsp16.9 (PDB access number 1GME) and *Pisum sativum* sHsp18.1 (PDB access number 5DS2) as templates [17] using the SWISS-MODEL [40]. Then, a dimer was modeled, taking advantage of the oligomeric structure prediction implemented in the SWISS-MODEL server. The absence of sequence similarity for the NTD and CTD prevented us from obtaining an adequate template to generate models for these regions.

For comparison, the AlphaFold *Pv*Nod22 model was downloaded from the EMBO database [28]. This model includes the whole protein and not just the ACS domain (Figure S3).

### 4.4. Cross-Linking

*Pv*Nod22 cross-linking with glutaraldehyde was performed to explore the oligomerization propensity. To start the cross-linking reaction, a solution of 0.05% (*v*/*v*) glutaraldehyde was added to 100 μg of recombinant *Pv*Nod22 suspended in 1 mL of PBS pH 7.5. The mixture was incubated at room temperature, and 40 μL aliquots were taken at 1, 2, 3, 4, 5 and 10 min. Reactions were stopped by quenching glutaraldehyde with 10 μL of 0.5 M Tris-HCl, pH 7.5. Proteins in each aliquot were analyzed by 15% (*w*/*v*) SDS-PAGE.

### 4.5. Affinity Chromatography

Surface-sterilized seeds of common bean (*Phaseolus vulgaris* L. cv. Negro Jamapa) were germinated on water-saturated paper towels in the dark at 28 °C for 2 days. Then, the bean seedlings were transferred to pots containing vermiculite and grown in a glasshouse with a controlled environment (26–28 °C, 16 h photoperiod). Plants were watered every other day with Fahraeus nutrient solution supplemented with 8 mM KNO_3_. Three weeks after emergence, plants were collected. A total of 1 g of fresh leaf-tissue was ground to a slurry in the presence of 2 mL of PBS, pH 7.5, containing 0.1% Triton X-100 supplemented with the protease inhibitor cOmpleteTM. After centrifugation at 20,000× *g* for 10 min, the protein concentration of the supernatant was determined by the BCA protein assay using BSA as the standard. A total of 5 mg of total protein suspended in 1 mL of PBS pH 7.5 supplemented with protease inhibitors was incubated for 30 min at room temperature (control) or at 42 °C. Additionally, to test the effect of hydrogen peroxide in the *Pv*Nod22 interaction, 1 mM hydrogen peroxide was added to a third sample.

Recombinant PvNod22 (5 mg) was mixed with 0.2 mL of the Ni-NTA resin equilibrated in PBS pH 7.5 by batch-absorption for 1 h at room temperature. The column was then washed with 20 volumes of PBS, pH 7.5, containing 20 mM imidazole and 10 mM β-mercaptoethanol followed by 20 volumes of PBS, pH 7.5. A total of 1 mg of leaf soluble protein (from control or treated samples) was added to the *Pv*Nod22-Ni-NTA resin for batch absorption and incubated for 30 min at room temperature. The column was washed using 20 volumes of PBS, pH 7.5, containing 20 mM imidazole. Putative *Pv*Nod22 ligands were eluted from the column with PBS and 300 mM imidazole. Imidazole from protein fractions was removed using a Sephadex G25 chromatography column. Protein profiles were analyzed on 15% (*w*/*v*) SDS-PAGE.

### 4.6. PvNod22 Antibody and Immunoblot Analysis

The peptide Ac-DQLELDMWRFRLPESTRC-OH coupled to keyhole limpet hemocyanin was used to generate a polyclonal antibody against *Pv*Nod22 (NeoMPS-Polypetide Labs., Zug, Switzerland). Protein samples were mixed with a Laemmli sample buffer (1:1), heated in boiling water for 2–5 min and electrophoresed with 15% (*w*/*v*) SDS-PAGE. Gels were transferred to Hybond ECL nitrocellulose membranes and subjected to immunoblot analysis using standard procedures.

### 4.7. Electron Microscopy

*Pv*Nod22 10 μL aliquots of freshly purified protein (100 μg/mL in PBS pH 7.5) were applied to glow-discharged (15 mA/30 s) copper formvar/carbon-coated microscopy grids (200 mesh) and incubated at 20 °C for 10 min. After two washes with double-distilled water, samples were negatively stained with 2% (*w*/*v*) uranyl acetate. The stain was removed by washing with double-distilled water, and grids were air-dried and analyzed using a Carl Zeiss Libra (Oberkochen, Germany) 120 transmission electron microscope (TEM) operated at 120 kV. Electron micrographs were recorded at a nominal magnification of 60,000× and analyzed with Image-Pro Plus V5.1 (Media Cybernetics, Rockville, MD, USA).

### 4.8. Dynamic Light Scattering

Dynamic light scattering measurements were carried out using a Zetasizer Nano ZSP (Malvern Instruments Ltd., Malvern, UK) at 633 nm with a scatter angle of 173°. *Pv*Nod22 samples were 1.8, 2.4, 3.2 and 13 µM, in PBS, pH 7.5. Samples were filtered with a sterile 0.22 µm membrane filter and centrifuged at room temperature at 15,000× *g* for 10 min before measurements. Translational diffusion coefficients were obtained by measurements of the decay rates of scattered light and the autocorrelation curves. The hydrodynamic radius (R_H_) values were calculated from particle diffusion coefficients (D) via the Stokes–Einstein equation: R_H_ = K_B_T/6πηD, where K_B_ is the Boltzmann′s constant, T is the temperature and η represents the viscosity of the solution. Typically, three runs with 11 scans of 10 s were obtained for each sample. The temperature was increased within a 25 °C to 60 °C range (2 °C per min). For every temperature, three independent measurements were performed. Data were analyzed using SEDPHAT/SEDFIT software 15.01b [41].

### 4.9. Isothermal Titration Calorimetry

A total of 1 mL of recombinant *Pv*Nod22 (~45 µM) was extensively dialyzed against PBS, pH 7.5, filtered through a sterile 0.22 µm membrane filter and degassed prior to loading into the syringe. ITC dissociation experiments were performed at 25 °C on an ITC200 instrument (Malvern Instruments, Malvern, UK). Titrations consisted of 10 injections of 2 μL of recombinant *Pv*Nod22 into the buffer solution of the cell′s instrument, with 180 s intervals between injections. Data correction was carried out by subtracting the signal obtained with the buffer. The heat flow induced by the dissociation of *Pv*Nod22 was recorded and analyzed by the instrument’s software. Three independent experiments with different protein batches were performed.

## Data Availability

The data that support the findings of this study are available on request from the corresponding author.

## Supplementary Materials

The following supporting information can be downloaded at: https://www.mdpi.com/article/10.3390/molecules27248681/s1, Figure S1: PvNod22 Sequence alignment; Figure S2: ACS-PvNod22 homology dimmer model; Figure S3: AlphaFold model; Figure S4: Glutaraldehyde crosslinking of PvNod22; Figure S5: Correlation curves of PvNod22 at different temperatures and concentrations.
