# Supplementary material for: The Common Bean Small Heat Shock Protein Nodulin 22 from Phaseolus vulgaris L. Assembles into Functional High-Molecular-Weight Oligomers
Source: Molecules. 2022 Dec 8;27(24):8681. doi: 10.3390/molecules27248681 (PMC9783675; doi:10.3390/molecules27248681)
Supplement: Supplementary file 1 [file molecules-27-08681-s001.zip › molecules-2015244-supplementary.pdf]

molecules

Supplementary Materials

The Common Bean Small Heat Shock Protein Nodulin 22 from *Phaseolus vulgaris* L. Assembles into Functional High-Molecular-Weight Oligomers

Arline Fernández-Silva [1,†], Fernando Lledías [2,†], Jonathan Rodríguez-López [2], Juan E. Olivares [2], Leidys French-Pacheco [1], Marcela Treviño [3], Carlos Amero [1,*] and Claudia Díaz-Camino [2,*]

1 LABRMN, Centro de Investigaciones Químicas, IICBA Universidad Autónoma del Estado de Morelos, Av. Universidad 1001, Colonia Chamilpa, Cuernavaca 62209, Mexico
2 Instituto de Biotecnología, Universidad Nacional Autónoma de México, Av. Universidad 2001, Colonia Chamilpa, Cuernavaca 62210, Mexico
3 School of Pure and Applied Sciences, Florida SouthWestern State College, Fort Myers, FL 33919, USA
* Correspondence: carlosamero@uaem.mx (C.A.); claudia.diaz@ibt.unam.mx (C.D.-C.); Tel.: +52-7773297997 (ext. 6043) (C.A.); +52-7773291666 (ext. 38138) (C.D.-C.)
† These authors contributed equally to this work.

-. Figure S1. *Pv*Nod22 Sequence alignment.

-. Figure S2. ACS-*Pv*Nod22 homology dimmer model.

-. Figure S3. AlphaFold model.

-. Figure S4. Glutaraldehyde crosslinking of *Pv*Nod22.

-. Figure S5. Correlation curves of *Pv*Nod22 at different temperature and concentrations.

```
PvNod22                DADVVVEEDPDCFRFVAETEG-IG-DVRAHTIEIHPGVTKIVVRDGGSVELS---------LDQLELDMWRFRLPESTRPELASAVFVDGELIVTVPKGHGEED 157
Hsp T.thalictroides     DADVLVEENPECFRFVVTTE-DVGHDVQTQTMEIYPGVTKIVLRGRNVLELD---------LDELELDLWRFRLPPSTKPELASAVYVDGELIVTVPKDMNMDE 146
Hsp A.chinensis        DADVSVVETADCFRFVVDTDDGIGGDVRAHTVEIYPGVTKIVVRGNDVVDLS---------VDELELDLWRFRLPEATVPERASAVYSEGELVVTVPKDANFDV 147
Hsp P.frutescens       DADVSIDETPESIRFTAATD-DISGDVRADAVEIYPGVTKIVIRGNGVVDLS---------GTEFELDLWRFRLPPSTRPELVSSAYEDGELIVTVPKGAATED 144
Hsp S.asiatica         DADVSIDETPGSLRFTAATD-DIGGGVWADAIEIHPGVTKIVVRGGGVADLS---------GTELELDLWRFRLPASTRPELASAAYDDGELVVTVPKGAATTE 145
Hsp C.melo             DADVSIVENSNCFRFVAETDG-VSEEFRAHTVQIHPGVIKVVVRQIGDLEST---------LDELEIDMWRFRLPETTLPELATAVFDGGKLIVTVPKREDA-- 148
Hsp S.tora             DADVSVEESPDFFRFVAETDG-IG-DVQAHTVEIHPGVTKIVVRESGSVELL---------LDELELDLWRFRLPESTRPELASAVFVDGELVVTVPKGEEELG 148
Hsp M.truncatula       DADVAVEEAADCFRFVAETEG-IG-EVRAHTVEIHPGVTKIVVRDGESLELS---------LDQLELDMWRFRLPDSTLPELASAVFVDGELIVTVPKGHEVQN 148
Nod22 C.melo           DADVLVEENTDCFRFIAVTDGNISDGVRAHAVEIHPGVIKIVVRENESLEMA---------IDELELDMWRFRLPETTRPELASAAFVDGELIVTVPKGNDEEN 148
Hsp G.australes        DADVAVEESPDCFKFVAETDGRIGDVVRAHTVEIHPGVTKIVIRSNSLVGFG--------LLDDLELDMWRFRLPETTRPELASAVYEDGELIVTVPKGGEVEN 152
Hsp T.cacao            DADVAVEESPDCFKFVAETDGRIGDVVRAHTVEIHPGVTKIVIRSNSLVDFS--------LLDDLELDMWRFRLPETTRPELASAVYEDGELIVTVPKGGEVEN 224
PBP S.suchowensis      DADVSVEENPDCFRFVAETDDDIGDV-RAHTVEIHPGVTKIVIRPNGYFDLS--------TLDDFELDMWRFRLPESTRPELASAVLAHGELIVTVPKGEE--G 141
PPT S.suchowensis      DAEVSVEENPDCFRFVAETDNNIGEV-RAHTVEIYPGVTKIVIRPNGYLELS--------PLADLELDMWRFRLPETTRPELASAVLADGELIVTVPKGEEVEE 201
Hsp P.sativum          --RVDWKETPEAHVFKADLPGLKKEEVK---VEVEDDRVL-QISGERSVEKEDKNDEWHRVERSSGKFLRRFRLPENAKMDKVKASMENGVLTVTVPK------ 95
Hsp T.aestivum          NARMDWKETPEAHVFKADLPGVKKEEVK---VEVEDGNVL-VVSGERTKEKEDKNDKWHRVERSSGKFVRRFRLLEDAKVEEVKAGLENGVLTVTVPKAEVKKP 142
                       .: :   *      * .             :::  .     :                  .    : ****   :  :  ..:    * * *****
```

Figure S1. *Pv*Nod22 Sequence alignment. Sequence alignment of ACS-*Pv*Nod22 with selected homologous protein obtained from BLAST protein analysis. Conserved residues with *Pv*Nod22 are colored in red. The asterisk indicate fully conserved residue, colon indicates conservation between groups of strongly similar properties and period show conservation between groups of weakly similar properties. PBP: Protein binding protein, PPT: Pre protein translocase

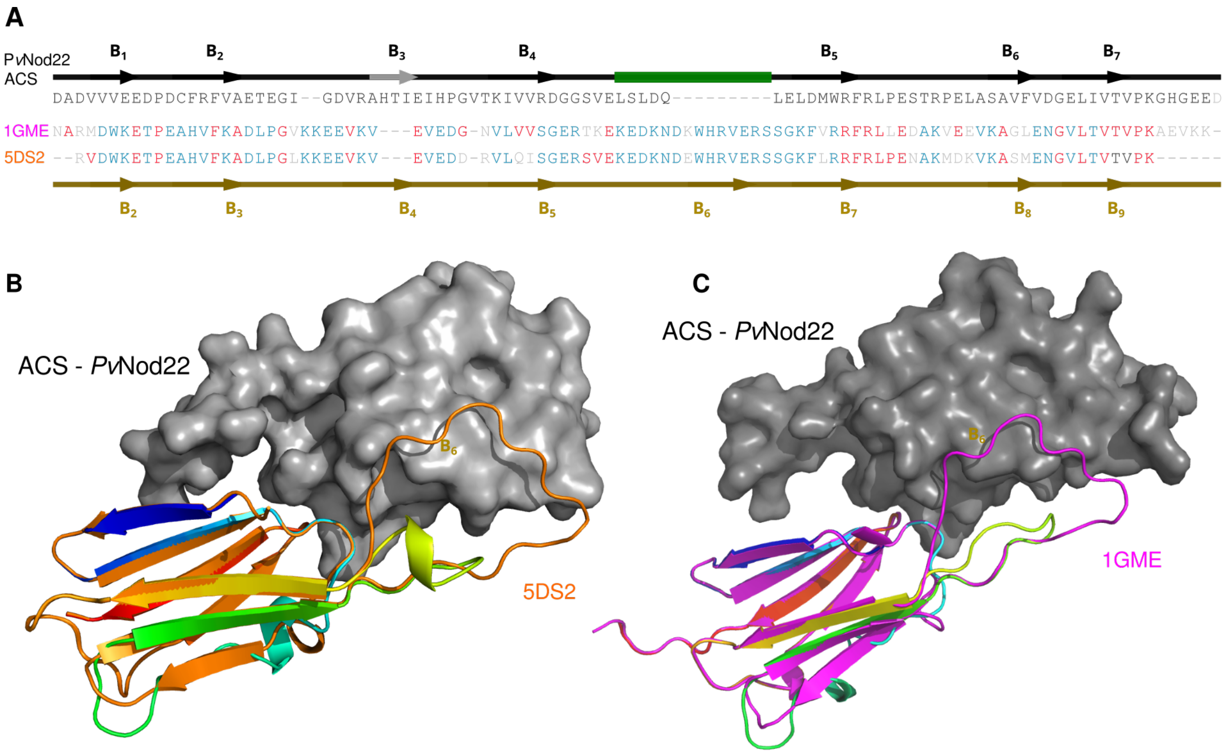

Figure S2. ACS-*Pv*Nod22 homology dimmer model. A) Sequence alignment of ACS-*Pv*Nod22 with *Triticum aestivum* sHsp16.9 (PDB access number 1GME) and *Pisum sativum* sHsp18.1 (PDB access number 5DS2). Conserved residues with *Pv*Nod22 are colored in red. Conserved residues between *Ta*sHsp16.9 *and* *Ps*sHsp18.1 are colored in blue. B) ACD dimmer homology model using the crystallographic structure of *Tas*Hsp16.9 (PDB code 5DS1) as template. The seven β-sheets predicted are shown multicolored. Template is shown in orange. C) ACD dimmer homology model using the crystallographic structure of *Pss*Hsp18.1 (PDB access number 5DS2) as template. The seven β-sheets predicted are shown multicolored. Template is shown in magenta.

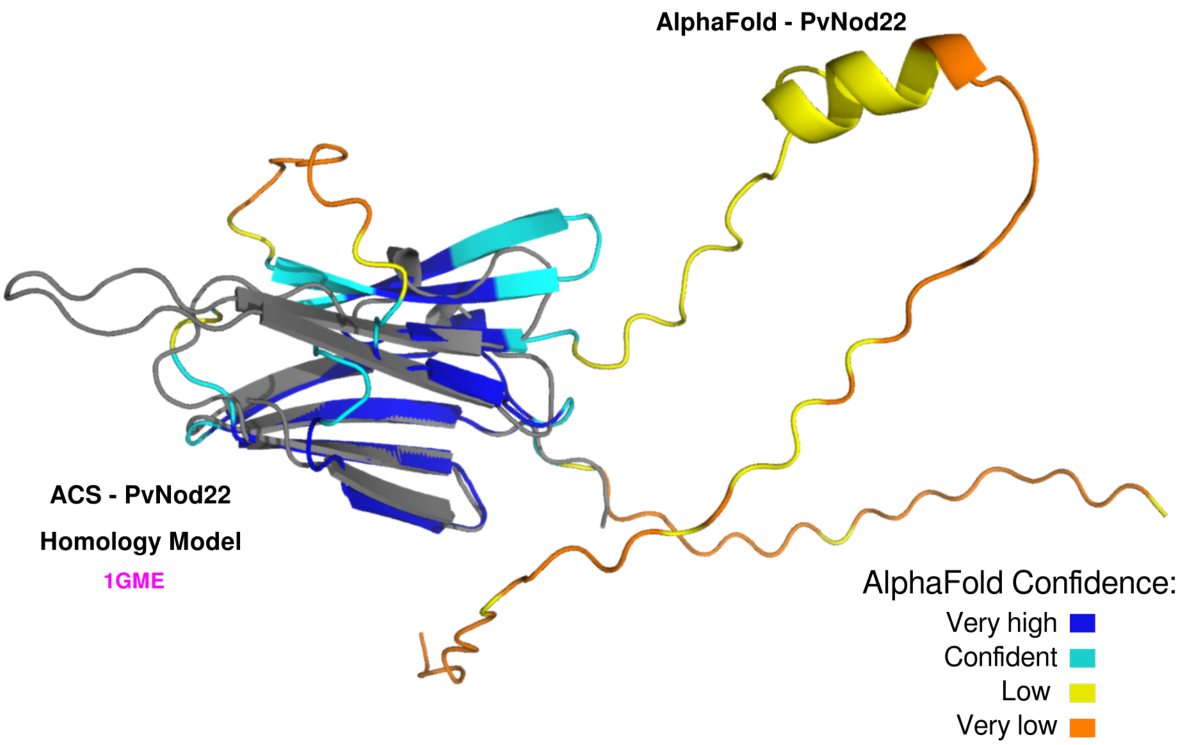

Figure S3. Superposition of ACS-*Pv*Nod22 Alphafold model and the homology model. Superposition of the ACS homology model using the crystallographic structure of *Tas*Hsp16.9 (PDB code 5DS1) and the Alphafold model downloads from the EMBO database. The Alphamodel include the N and C domains. Alphamodel color coded according to model confidence, Very high (pLDDT > 90) dark blue; Confident (90 > pLDDT > 70) blue; Low (70 > pLDDT > 50) yellor; Very low (pLDDT < 50) orange. The RMSD calculated between the confident regions of AlphaFold model and the homology model is 0.49 A.

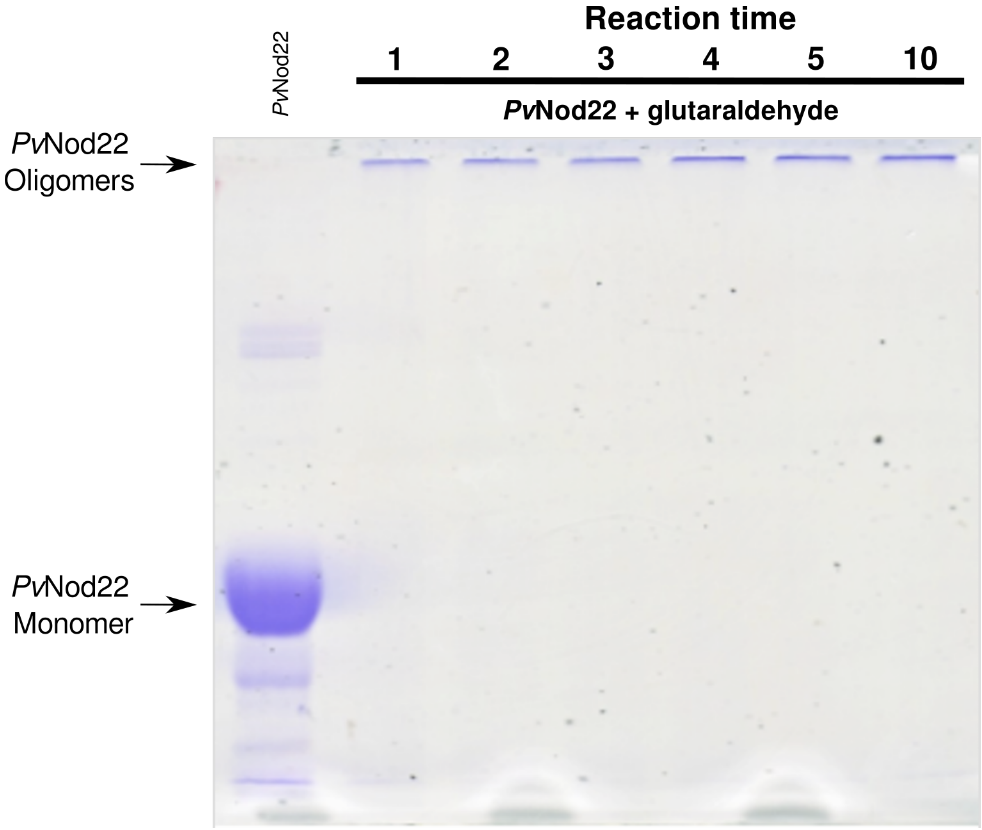

Figure S4. Glutaraldehyde crosslinking of *Pv*Nod22 for the identification of quaternary protein structure. SDS–PAGE, 6% Laemmli of *Pv*Nod22 recombinant protein (1 mg/mL) treated with glutaraldehyde 0.05%. (v/v) for limited times (min). Lane C. Purified recombinant *Pv*Nod22 untreated with glutaraldehyde. Lanes 1-5 and 10, min after glutaraldehyde treatment. The figure show that the proteins form high molecular weight complex are immediately formed after glutaraldehyde addition.

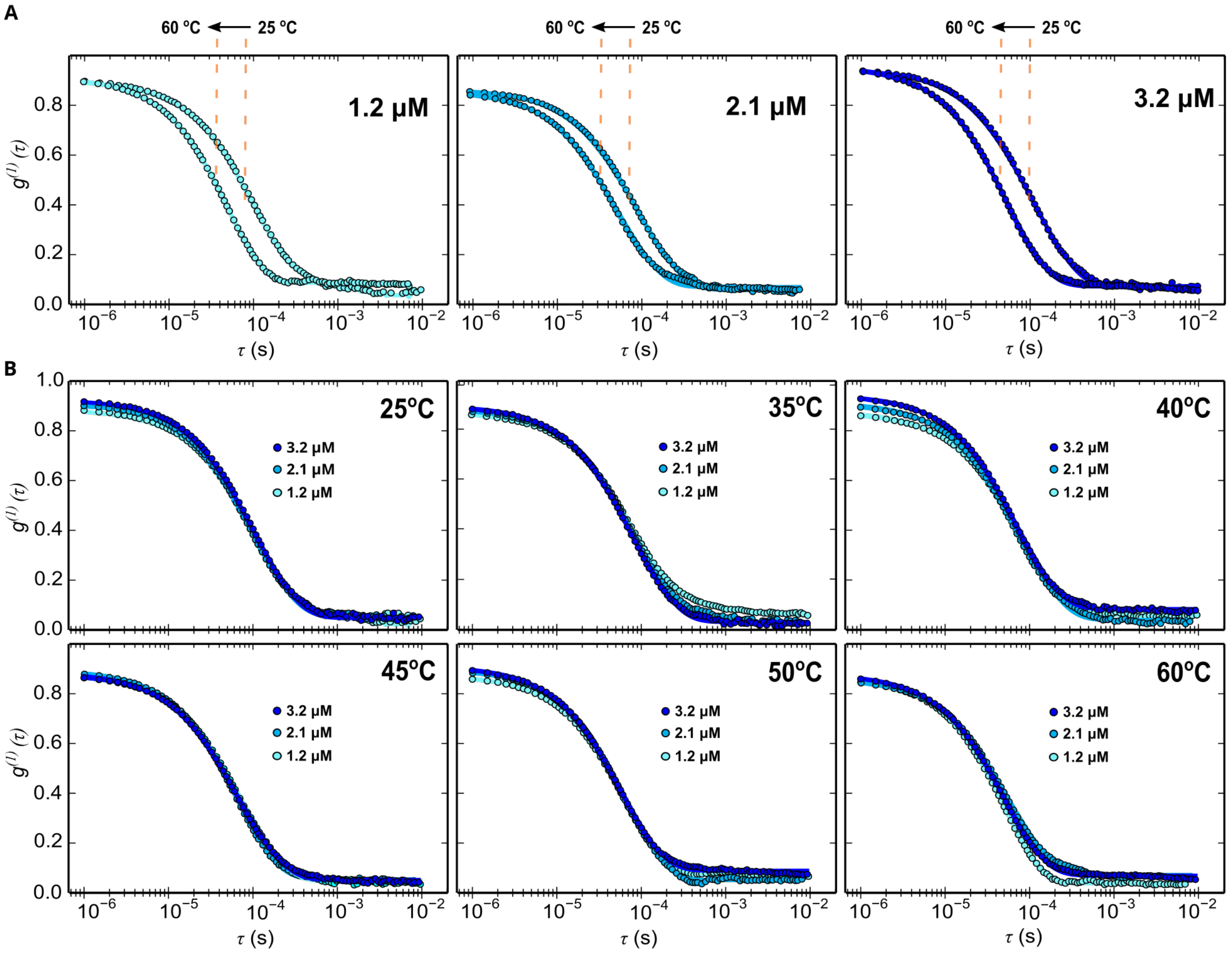

Figure S5. Correlation curves of *Pv*Nod22 at different temperature and concentrations. A) Correlation curves at 1.3, 2.1 and 3.2 μM of *Pv*Nod22 at different temperatures. Discontinuous lines show the movement from left to right observed with the temperature increased from 25 to 60°C. B) Correlation curves at 25, 35, 40, 45 ,50 and 60°C at different concentration. Light scattering measurements were carried out using a Malvern Zetasizer Nano ZSP at 633 nm with a scatter angle of 173°. *Pv*Nod22 samples were previously diluted in TBS. The figure show that the concentrations of *Pv*Nod22 do not have relevant effect on the molecular size of the oligomers.
